# The effect of different depths of medial heel skive on plantar pressures

**DOI:** 10.1186/1757-1146-4-S1-O10

**Published:** 2011-05-20

**Authors:** Daniel R Bonanno, Cheryl Y Zhang, Rose C Farrugia, Matthew G Bull, Anita Raspovic, Adam R Bird, Karl B Landorf

**Affiliations:** 1 Department of Podiatry, Faculty of Health Sciences, La Trobe University, Bundoora, Victoria, 3086, Australia; 2 Musculoskeletal Research Centre, Faculty of Health Sciences, La Trobe University, Bundoora, Victoria, 3086, Australia

## Background

Foot orthoses are often used to treat lower limb injuries associated with excessive pronation. There are many orthotic modifications available for this purpose, with one being the medial heel skive. However, empirical evidence for the mechanical effects of the medial heel skive is limited. This study aimed to evaluate the effect that different depths of medial heel skive have on plantar pressures.

## Methods

Thirty healthy adults aged over 18 years with flat feet and no current foot pain or deformity participated in this study. Using the in-shoe Pedar^®^ system, plantar pressure data were collected for the heel, midfoot and forefoot while participants walked along an 8 metre walkway wearing a standardised shoe. Experimental conditions included the following 4 orthotic variants: (i) no heel skive, (ii) 2 mm heel skive, (iii) 4 mm heel skive and (iv) 6 mm heel skive.

## Results

Compared to the foot orthoses with no heel skive, statistically significant increases in peak pressure were observed at the medial heel – there was a 15% increase (p = 0.001) with the 4 mm skive and a 29% increase (p < 0.001) with the 6 mm skive. No significant change was observed with the 2 mm heel skive. With respect to the midfoot and forefoot, there were no significant differences between the orthoses.

## Conclusions

These findings indicate that a medial heel skive of 4 or 6 mm can increase peak pressure under the medial heel in asymptomatic flat-footed individuals. Plantar pressures at the midfoot and forefoot were not affected by a medial heel skive of 2, 4 or 6 mm. These findings provide some evidence for the effects of the medial heel skive orthotic modification.

